# The Effect of the TiO_2_ Anodization Layer in Pedicle Screw Conductivity: An Analytical, Numerical, and Experimental Approach

**DOI:** 10.3390/bioengineering11070634

**Published:** 2024-06-21

**Authors:** Pedro Fonseca, Márcio Fagundes Goethel, João Paulo Vilas-Boas, Manuel Gutierres, Miguel Velhote Correia

**Affiliations:** 1 Porto Biomechanics Laboratory, Faculty of Sports, University of Porto, 4200-450 Porto, Portugal; 2 Faculty of Engineering, University of Porto, 4200-465 Porto, Portugal; 3 Faculty of Sports, University of Porto, 4200-450 Porto, Portugal; 4 Centre of Research, Education, Innovation and Intervention in Sport, Faculty of Sport, University of Porto, 4200-450 Porto, Portugal; 5 Faculty of Medicine, University of Porto, 4200-319 Porto, Portugal; 6 Institute for Systems and Computer Engineering, Technology and Science, 4200-465 Porto, Portugal

**Keywords:** neuromonitoring, pedicle screw, electrical resistance, conductivity, oxide layer

## Abstract

The electrical stimulation of pedicle screws is a technique used to ensure its correct placement within the vertebrae pedicle. Several authors have studied these screws’ electrical properties with the objective of understanding if they are a potential source of false negatives. As titanium screws are anodized with different thicknesses of a high electrical resistance oxide (TiO_2_), this study investigated, using analytical, numerical, and experimental methods, how its thickness may affect pedicle screw’s resistance and conductivity. Analytical results have demonstrated that the thickness of the TiO_2_ layer does result in a significant radial resistance increase (44.21 mΩ/nm, for Ø 4.5 mm), and a decrease of conductivity with layers thicker than 150 nm. The numerical approach denotes that the geometry of the screw further results in a decrease in the pedicle screw conductivity, especially after 125 nm. Additionally, the experimental results demonstrate that there is indeed an effective decrease in conductivity with an increase in the TiO_2_ layer thickness, which is also reflected in the screw’s total resistance. While the magnitude of the resistance associated with each TiO_2_ layer thickness may not be enough to compromise the ability to use anodized pedicle screws with a high-voltage electrical stimulator, pedicle screws should be the subject of more frequent electrical characterisation studies.

## 1. Introduction

Intraoperative neuromonitoring allows the continuous supervision of the neurophysiological parameters of the patient to ensure that no lasting negative effects result from a surgical intervention [1]. Among the techniques pertaining to pedicle screw insertion is triggered electromyography (tEMG), which consists in the electrical stimulation of the pedicle hole or the implanted pedicle screw itself, to elicit a muscle electric potential Calancie et al. [2] introduced this technique in an animal model and later demonstrated it in humans [3]. Its working principle is that the application of a small electrical current will only stimulate the surrounding nerve structures and elicit a muscle potential in the enervated muscles if a low-resistance path is present. This should only occur in the presence of a pedicle wall or vertebral body breach, as in other cases the bone structures will act as insulators. 

While the electrical probing of the pilot hole is recommended [4], since the insertion of the pedicle screw may generate further removal of pedicular and vertebral mass, the stimulation of the pedicle seems to gather more interest. Ideally, this method would enable the verification of the goodness of pedicle screw insertion without the need for imaging confirmation. However, current evidence has demonstrated a low sensitivity and high specificity of tEMG [5], indicating a high rate of false negatives. Anderson et al. [6] suggested that this could be related to high pedicle screw resistance, which led researchers to investigate pedicle screws’ electrical properties in uni- and polyaxial titanium screws [6,7,8,9], as well as in those coated with hydroxyapatite [10,11]. Nevertheless, testing methods are still too heterogeneous, and results difficult to compare. In some cases, the results even seem contradictory, as with Davis et al. [11], who reported that hydroxyapatite creates a strongly insulating layer around the pedicle screw, with resistance greater than 1 MΩ. Still, Davis et al. [10] did not find such a strong resistive effect for this type of coating with a different set of screws and experimental conditions. This suggests that coating may significantly alter the ability to conduct electricity through the pedicle screws, particularly if they have insulating properties.

A titanium alloy, specifically Ti-6Al-4V, is currently the most common material used in pedicle screw manufacturing, as it presents good mechanical properties and high resistance to chemical and biological corrosion. This resistance comes from the 5 to 25 nm naturally forming titanium oxide (TiO_2_) layer that develops on the titanium surface when in contact with free oxygen molecules [12]. This layer can be thickened by anodization procedures, allowing TiO_2_ up to 250 nm to be formed [12,13] and due to light interference between the oxide layer and the underlying titanium, present a wide range of apparent colourations [12,14]. These have been reported as useful for aesthetical [15,16] and technical purposes, such as facilitating the surgeon to differentiate between screw diameters or other features [12,17]. This colouration can also be used as an indirect measure of the TiO_2_ layer thickness [16]. Despite all these advantages, TiO_2_ is a semiconductor material with a high electrical resistance at room temperature [18]. Indeed, Donohue et al. [4] speculated that the differences in conductivity among titanium screws could be related to their oxide layer properties. However, to our knowledge, no study has delved into the effect of different TiO_2_ layer thicknesses in pedicle screws and how it may affect their ability to conduct electrical current, thus warranting a need to further research this matter.

As such, this study aimed to investigate the extent to which different layer thicknesses may compromise the pedicle screw’s overall electrical conductivity. We selected electrical conductivity as our main parameter for comparison, as this is an intrinsic material property. We also considered the pedicle screws as a layered composite (i.e., a material composed of two distinct materials bonded as layers), meaning that the overall screw conductivity was calculated by considering the contribution of the resistance of both materials.

We hypothesised that pedicle screws with different TiO_2_ layer thicknesses would present distinct electrical conductivities. To test our hypothesis, we performed a series of analytical, numerical, and experimental studies. The analytical approach intends to provide an indication of how much the radial resistance of the TiO_2_ layer will impact conductivity. The numerical approach further builds on this by adding geometrical complexity, and finally, the experimental approach provides real-life validation of the previously obtained mathematical results.

## 2. Materials and Methods

### 2.1. Analytical Approach

**Figure 1:**
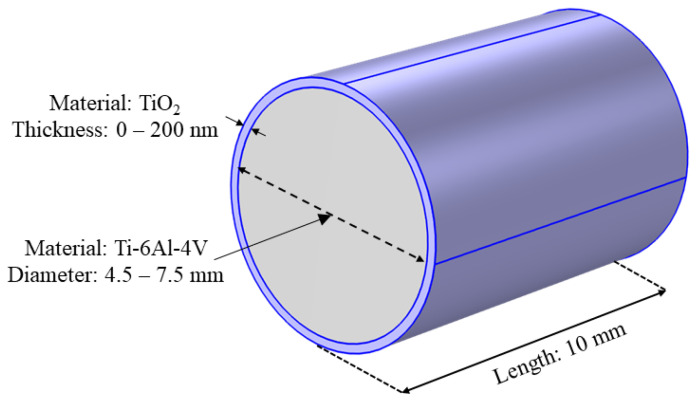
Geometrical model used for the numerical analysis of the effect of different TiO_2_ layer thicknesses on a Ti-6Al-4V core. TiO_2_ layer representation is not to scale.

To obtain the reference electrical conductivity of the anodized pedicle screws, analytical calculations based on a first-principles approach were performed using a simplified geometrical model. This model consisted of a 10 mm length Ti-6Al-4V cylinder with diameters of 4.5, 5.5, 6.5, and 7.5 mm, surrounded by a TiO_2_ layer with thicknesses ranging from 0 to 200 nm, with 25 nm increments. A 5 nm TiO_2_ thickness was also considered, as this is the minimum naturally occurring thickness of TiO_2_ [12]. A representation of the geometrical model and its dimensions is presented in Figure 1.

The radial resistance of the TiO_2_ annulus was calculated since the TiO_2_ layer surrounds the entirety of the Ti-6Al-4V core, and the electrical current must cross it to enter the core. Then, the Ti-6Al-4V core resistance and its contribution to the overall model resistance and conductivity was also calculated.

The Drude-Lorentz Free Electron Theory [19] was used to first estimate the TiO_2_ layer conductivity. According to this theory, electrical conductivity (σ) is dependent on the number of free electrons per unit of volume (*n*), the electron charge (*e*) and mass (*m*), and the relaxation time (τ), as expressed in Equation (1):

(1) $$\sigma =\frac{n\cdot e^{2}\cdot \tau }{m}$$

This equation can be simplified if the mobility of electrons $\left(\mu \right)$, presented in Equation (2), is considered [19]:

(2) $$\mu =\frac{e}{m}\cdot \tau$$

If both equations are conjugated, the electrical conductivity can be expressed by Equation (3) as

(3) σ=n·e·μ

The TiO_2_ conductivity was calculated with values retrieved from the scientific literature at room temperature (approximately 300 K). A free electron concentration of 10^14^ cm^−3^, an electron charge of 1.6 × 10^−19^ C, and an electron mobility in thin films of 0.1 cm^2^/V·s were considered [20,21,22]. The Ti-6Al-4V core electrical conductivity was obtained from COMSOL 6.1 software (σ = 0.581 MS/m, at 25.0 °C).

The microscopic formulation of Ohm’s Law, presented in Equation (4), was used for further calculations. This equation demonstrates that the current density (*J*) in an object is dependent on its conductivity (σ) and electric field (*E*):

(4) J=σ⋅E

As the current density is equivalent to the current (*I*) by unit of area (*A*), and the electrical field is dependent on the potential difference (*V*) over a given length (*l*), we have the equivalence denoted by Equation (5):

(5) $$\frac{I}{A}=\sigma \cdot \frac{V}{l}$$

Considering electrical resistance (*R*) as the quotient between electrical potential (*V*) and current (*I*) we can derive resistance as

(6) $$R=\frac{l}{\sigma \cdot A}$$

This equation allows the calculation of a material’s electrical resistance (R), which is dependent on the sample’s electrical conductivity (σ), its length (*l*), and cross-sectional area (*A*). It can also be arranged as per Equation (7) to allow the calculation of electrical conductivity:

(7) $$\sigma =\frac{l}{R\cdot A}$$

While the calculation of the core Ti-6Al-4V axial resistance ($R_{Core}$) was performed with Equation (6), the TiO_2_ layer radial resistance was obtained from integration of Equation (5) as a function of its radius (*r*):

(8) $$\frac{I}{A}=\sigma \cdot \frac{dV}{dr}$$

(9) $$\frac{dV}{I}=\frac{1}{\sigma \cdot A}dr$$

(10) $$R=\int \frac{1}{\sigma \cdot \pi \cdot r^{2}\cdot l}\;dr$$

As the TiO_2_ layer limits are defined by the Ti-6Al-4V core radius (*r_i_*) and the model’s outer radius (*r_o_*), the defined integral between these limits results in the TiO_2_ layer resistance:

(11) $$R=\int_{r_{i}}^{r_{o}}\frac{1}{\sigma \cdot \pi \cdot r^{2}\cdot l}\;dr$$

(12) $$R=\frac{1}{\sigma \cdot 2\pi \cdot l}\cdot ln\left(\frac{r_{o}}{r_{i}}\right)$$

Once both the Ti-6Al-4V core and the surrounding TiO_2_ layer resistances were obtained, the total model resistance was calculated considering these resistances as being in parallel. Total conductivity was calculated using Equation (7), considering the model’s cross-sectional area as that obtained from the outer radius.

### 2.2. Numerical Approach

An electrical currents simulation experiment was conducted using COMSOL 6.1 (COMSOL AB, Stockholm, Sweden) software. A 3D digital model of a commercial screw from Spine Implantes (Rio Claro, São Paulo, Brazil), with a 6.5 mm diameter and 40 mm thread length, was used.

**Figure 2:**
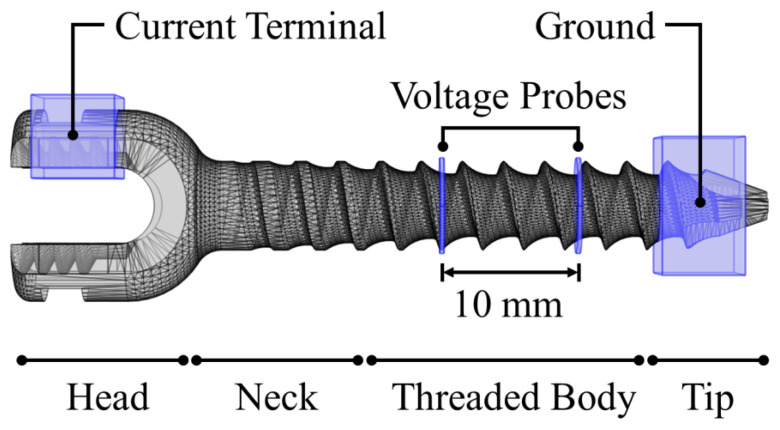
Representation of the pedicle screw 3D model and the domains corresponding to the current terminal, ground, and voltage probes.

To simulate a four-probe setup, a 6 × 11 × 5 mm domain was placed over one of the screw’s head edges as a current terminal, while another with 6 × 10 × 10 mm was placed at the tip, acting as ground. Two cylindrical domains with 0.3 mm thickness and 7.0 mm diameter were placed 10 mm apart from each other, at the centre of the screw’s threaded body, as voltage probes. The volume occupied by the screw in these structures was removed by Boolean subtraction. A representation of the numerical model is presented in Figure 2.

The pedicle screw domain material was modelled as the default COMSOL Ti-6Al-4V ELI Grade 23 titanium alloy (σ = 0.581 MS/m, at 25.0 °C), while the current terminal, ground, and voltage probe domains were modelled as copper (σ = 59.98 MS/m). All screw surface boundaries were modelled as TiO_2_, with the conductivity obtained from the analytical calculations. After a mesh convergence study, the model was meshed once using a physics-controlled setting.

The voltage drop between the probes was measured at a constant current of 10 mA, and a parametric sweep was used to test the effect of TiO_2_ thickness. The same values of thickness used in the analytical calculations were used. The electrical resistance in the measurement region was calculated using Ohm’s Law, while electrical conductivity was calculated using Equation (7). The cross-sectional area was measured at the mid-point between the voltage probes (*A* = 24.50 mm^2^), while the length was the distance between the voltage leads (*l =* 10 mm). The terminal voltage and resistance were also registered as representatives of the entire screw voltage drop and resistance, respectively.

### 2.3. Experimental Approach

An experimental setup was devised to measure the electrical resistance of a set of eight solid uniaxial titanium alloy (Ti-6Al-4V, ASTM F136) pedicle screws from the Spinelock set (Spine Implantes, Rio Claro, São Paulo, Brazil). All screws were anodized by the manufacturer according to the ABNT NBR ISO 15408-2:2015 standard [23] using sodium bicarbonate as the anodization electrolyte. 

The screws were categorised into four groups according to their nominal thread outer diameter, with groups ranging from a single screw to three. All screws in each group had the same diameter, anodization voltage, and apparent anodic colour, although presenting different lengths. A name was devised to identify each screw model according to their apparent Munsell hue and chroma notation and the corresponding anodization voltage [15]: Dark Brown (DB, Group I), Shallow Blue (SB, Group II), Bright Golden (BG, Group III), and Dark Green (DG, Group IV). A summary of the screws, their physical characteristics, and anodization voltages is presented in Table 1. The theoretical TiO_2_ anodic layer thickness was calculated considering a 2.0 nm/V growth rate reported by Zaniolo et al. [14].

Each screw was cleaned with isopropyl alcohol to remove any grease or dirt from its surface. A B&K Precision 1672 (B&K Precision, Yorba Linda, CA, USA) power supply and a 1 kΩ trimmer were connected in series with the pedicle screw under test, with the anode connected to one of the screw’s head sides and the cathode to its tip, in an experimental replication of Figure 2. The trimmer was adjusted so that a constant direct current of 10 mA was applied to the screw, which was confirmed with an Agilent U1251B (Agilent, Santa Clara, CA, USA) ammeter. Then, the voltage drop was measured with an Agilent 34405A (Agilent, Santa Clara, CA, USA) over a nominal 10 mm section of each screw specimen. This procedure was performed five times, after a 5-s settling period, using the “capsule” coupling method described by Fonseca et al. [7].

Electrical conductivity was calculated using Equation (7). The cross-sectional area was calculated from the 3D digital model of the DB2 screw (the same used in the numerical analysis), with the remaining screw area being calculated by isotropic scaling of the digital model. The length was that corresponding to the distance between the voltage leads. Since their application was a manual process prone to inaccuracy, their effective distance was verified with a calliper. Five measurements of this distance were performed and the average value was used for the calculations.

The total electrical resistance of the screws was measured with an Agilent U1251B multimeter (Agilent, Santa Clara, CA, USA). Alligator clips were placed at the head and tip of the screw, and the resistance value was recorded after a 5-s settling period. This procedure was performed five times for each screw. The measurement leads resistance was subtracted from all measurements. These procedures were performed on a single day at a room temperature of 25.0 °C.

### 2.4. Statistical Analysis

Statistical analysis was performed on the electrical conductivity obtained from the experimental study of each analysed pedicle screw after 1000-sample bootstrapping. Data distribution was verified through the Kolmogorov–Smirnov test, after which an analysis of variance (ANOVA) was performed to analyse the effect of group and individual screw conductivity. Post-hoc pairwise analysis was conducted with Bonferroni correction. All these procedures were performed using IBM SPSS Statistics v. 29.0 (IBM Corp., Armonk, NY, USA) with a significance level of α = 0.05. The effect size was calculated with G*Power 3.1.9.7 (University of Kiel, Kiel, Germany) and expressed as the Cohen’s *d* value (Cohen, 1992), interpreted as small (>0.2), moderate (>0.5), or large (>0.80).

## 3. Results

### 3.1. Analytical Approach

**Figure 3:**
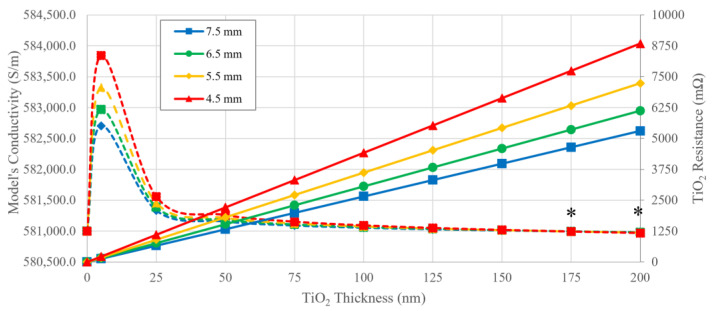
Resistance values (solid lines) of the TiO_2_ layer for different thicknesses and for each model’s diameter and the resulting model’s conductivity (dashed lines). An asterisk (*) denotes the layer thickness with a conductivity below that of uncoated Ti-6Al-4V.

The calculation of the TiO_2_ conductivity using Equation (3) results in a value of 0.16 mS/m for a thin film. This was used to calculate the resistance of the TiO_2_ layer around the geometrical model with different diameters and the model’s overall conductivity. In Figure 3, the effect of the increased TiO_2_ thickness on its electrical resistance and the resulting model conductivity for different diameters are presented.

In the absence of a TiO_2_ layer, the model’s electrical resistance and conductivity are the same as those of Ti-6Al-4V, and dependent on this material’s diameter and length. The oxide, on the other hand, presents a linear increase in resistance, which is attributed to the uniform increase in its thickness. The rate of TiO_2_ resistance increase is dependent on the model’s diameter, with smaller diameters being associated with higher rates of resistance: 26.53 mΩ/nm (Ø 7.5 mm), 30.61 mΩ/nm (Ø 6.5 mm), 36.17 mΩ/nm (Ø 5.5 mm), 44.21 mΩ/nm (Ø 4.5 mm). This results in larger resistance differences between the models for thicker TiO_2_ layers.

On the other hand, the model’s conductivity seems to be more affected by smaller TiO_2_ thickness layers. With the addition of a 5 nm layer, the conductivity increases to values higher than that of uncoated Ti-6Al-4V, and then follows an asymptotic reduction towards this value. While the TiO_2_ layer seems to improve the conductivity of the geometrical models, especially in those with smaller diameters, a layer thickness greater than 150 nm presents a drop in conductivity to values below those of the uncoated Ti-6Al-4V in all models.

Despite the TiO_2_ layer resistance ranging between 0.13 and 8.84 Ω with increased layer thickness, the model’s total resistance has a more modest variation. For all models’ diameters, the total resistance increases by 1.0 µΩ or less with the increase of the TiO_2_ layer, which results in an average model resistance of 0.39 mΩ (Ø 7.5 mm), 0.52 mΩ (Ø 6.5 mm), 0.72 mΩ (Ø 5.5 mm) and 1.08 mΩ (4.5 mm).

### 3.2. Numerical Approach

To add geometrical complexity to the study, a digital representation of a 6.5 mm diameter pedicle screw was used as a model in this approach. While the terminal resistance (i.e., the entire pedicle screw model resistance) increases with the TiO_2_ layer thickness at a linear rate of 125 mΩ/nm, the section of analysis does not present such linear behaviour. The section resistance presents changes under 1.0 µΩ as the TiO_2_ layer thickens. As reported in Table 2, the section resistance obtained in this analysis is greater than that calculated using analytical methods for a model with a similar nominal diameter. The numerical model section conductivity, on the other hand, is smaller, especially when compared with uncoated Ti-6Al-4V. 

**Figure 4:**
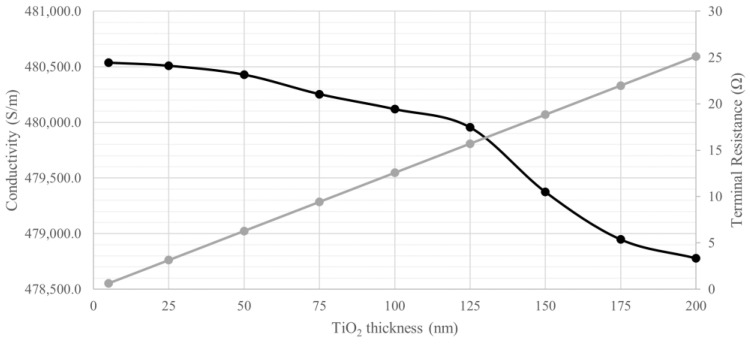
Electrical conductivity (black) and total resistance (grey) variation of the pedicle screw according to the TiO_2_ thickness from 5 to 200 nm.

As depicted in Figure 4, the section conductivity decreases at an almost linear rate between 5 and 125 nm, with an average of −4.7 ± 2.4 S.m^−1^/nm. After this layer thickness, the conductivity presents a sharper decrease, averaging −15.7 ± 8.3 S.m^−1^/nm. This rate of conductivity loss is greater than that observed with the simplified geometry of the analytical approach.

### 3.3. Experimental Approach

Differences between screws were found (F_(7,7992)_ = 4651.64, *p* < 0.001, $\eta_{p}^{2}$ = 0.803) in terms of their electrical conductivity, which are, along with the measured resistance, depicted in Table 3.

While within Group I, only DB2 differs from the other specimens (DB1: *p* < 0.001, *d* = 1.74; DB3: *p* < 0.001, *d* = 1.68), all screws in Group III present differences between them. BG1 has a smaller conductivity than BG2 (*p* < 0.001, *d* = 1.77) and BG3 (*p* < 0.001, *d* = 2.68), with BG3 featuring the highest conductivity of the group, differentiating itself also from BG2 (*p* < 0.001, *d* = 2.01). SB and DG, while sole specimens of their group, also present differences with the rest of the screws analysed (*p* < 0.001). SB does not significantly differ from BG3.

When pooling the screws by anodization voltage, a significant difference between groups was found (*F*_(3,7996)_ = 4510.3, *p* < 0.001, $\eta_{p}^{2}$ = 0.629). Post-hoc analysis revealed a decrease in the conductivity associated with increasing TiO_2_ layer thickness. Considering the group conductivity, the highest value was found in Group I (σ = 0.556 ± 0.037 MS/m), which is the one with the thinnest theoretical TiO_2_ layer. Each group presents a significant decrease in conductivity in comparison to the previous group (*p* < 0.001). Group II conductivity is 1.3% smaller than that of Group I (*d* = 0.42), with a further decrease of 2.9% being observed in Group III versus Group II (*d* = 1.29), and finally, a decrease of 7.7% observed in Group IV versus Group III (*d* = 2.89).

Total screw resistance seems to present the expected results within the group, with longer screws resulting in higher resistance. The same cannot be said regarding the inverse relationship between resistance and diameter. The screws DG, DB2, and BG1 have the same length but decreasing diameters, which should have resulted in increased resistance. However, increased resistance is found in DB2, BG1, and DG, in the same order as the decreasing conductivity.

## 4. Discussion

In this study, an analytical, numerical, and experimental investigation of pedicle screws electrical properties was performed. Every model was considered to comprise a thick inner Ti-6Al-4V layer and a very thin coating layer of TiO_2_. Since the two materials cannot be separated during pedicle screw electrostimulation, they were considered as a composite. This means that, although having individual electrical properties, the studied models (and pedicle screws by extension) have composite electric properties as well. 

These properties can differ from those of the individual materials, especially taking into consideration the testing methods. Electrical conductivity was chosen as the main comparator in this study and was established as a constant for both the Ti-6Al-4V and TiO_2_ materials. However, the calculation of the composite material conductivity using diverse approaches yielded different results. As it becomes apparent from a deeper analysis of the results, regardless of the calculation approach, the screw area and geometry seem to have a significant impact.

### 4.1. Analytical Approach

Titanium oxide presents several polymorphs, with anatase and rutile being the most common crystalline allotropes [12]. The scientific literature provides a wide range of electrical conductivity values for these structures, with values ranging from 10^−11^ S/m [24] to 10 S/m [18], depending on their crystalline structure and thickness.

The naturally occurring TiO_2_ layer formed by exposure to air is amorphous, as is the layer obtained from anodization at low voltages [13]. However, the anodic layer characteristics are susceptible to changes depending on the oxidation procedure [13], electrolyte temperature [25], and even the applied voltage [12]. Annealing can also promote the rearrangement of the crystalline structure, with a temperature of 350 °C allowing the conversion to anatase [13,26] and higher than 800 °C resulting in rutile [20]. Microscopic studies have also identified anatase as the first forming crystals in anodic layers, especially those obtained at higher anodization voltages [12,14,27,28]. As such, adhering to the specific properties of a given polymorph is challenging, especially when the thin film and bulk properties differ [29,30,31] and when manufacturing procedures are not clearly known. By resorting to analytical calculations based on first principles, the magnitude of the TiO_2_ electrical properties can be known.

In this study, the electrical conductivity of the material was calculated using the Drude-Lorentz equation, considering the electron concentration and mobility of the thin film. While the carrier concentration used is inferior to values found in other sources for anatase [32], this compensates for the worse charge transmission that occurs in amorphous TiO_2_ [13]. Increased carrier concentrations are usually found in the presence of doping elements, which also result in alterations in the mean relaxation time and, therefore, the electron mobility [33]. A low mobility is also a feature of thin layers due to the reduced mean free path available for the electrons [31]. This resulted in a calculated TiO_2_ conductivity of 0.16 mS/m, which is in the semiconductor band range [34] and in agreement with the conductivity range presented by Prasad et al. [35] for non-doped and non-amorphous TiO_2_ thin layers. Since this value was obtained from a first-principles approach, it reflects the ideal TiO_2_ conductivity, not contemplating the resistivity due to scattering effects and impurities [36], which could have a deleterious effect on its conductivity.

The equivalent circuit for oxide-coated titanium generally presents these materials arranged as series resistances with parallel capacitors [37,38]. For a fully coated material, this is a logical representation, as any external electrical current would have to cross the coating before reaching the core. Since this study was performed with direct current, and given the low capacitance of TiO_2_ (<4.5 pF) [39], it is reasonable to expect that these capacitors will quickly become open circuits, leading all the current through the resistors. However, a series arrangement would require the sum of the TiO_2_ and Ti-6Al-4V resistances, resulting in a larger model resistance and low conductivity, which is not in line with the results of experimental studies [7,8,9,40].

If the materials’ resistances are arranged in parallel, this would explain why, despite the increase in TiO_2_ resistance, the conductivity was better than that of the uncoated Ti-6Al-4V up to 175 nm. In a parallel arrangement, the Ti-6Al-4V core will provide the main current conduit, and for a small TiO_2_ thickness where the resistance is still low, TiO_2_ provides an alternative current path. At a larger TiO_2_ thickness, its resistance becomes too high, which would indicate that as it tends to infinity, the total resistance and conductivity would match those of the uncoated Ti-6Al-4V. A parallel resistance arrangement and a lower-resistance material acting as the dominant current path are generally associated with multi-layered materials [41,42].

The reports available for pedicle screw electrical resistance, however, do not seem to indicate a trend towards Ti-6Al-4V resistance and conductivity. In fact, Limthongkul et al. [8] reported conductivities of 0.439 MS/m and 0.418 MS/m in pedicle screws of 6.25 to 7.5 mm diameter, respectively. These values are in agreement with those of Davis et al. [11], who reported an average conductivity of 0.421 MS/m for 6.5 mm diameter screws, and with Fonseca et al. [7], who reported 0.419 MS/m for 4.5 mm screws and 0.442 MS/m for 5.0 mm screw diameter. This apparent disagreement may be related to the experimental conditions since most studies have used alligator clips as current and ground terminals. Their pressure may be enough to puncture the TiO_2_ layer and create a low-resistance path to the inner Ti-6Al-4V core of the screw. This results in the current source being simultaneously in contact with the TiO_2_ layer and the Ti-6Al-4V core, thus creating a parallel circuit. However, this does not explain why the results of Limthongkul et al. [8], using a non-penetration apparatus, were in the same range of values as in other experimental studies.

By considering the two materials’ resistances as being in parallel, the changes in conductivity were mainly caused by the increase in the model’s cross-sectional area, rather than an effective resistive effect. This explains the reduction in conductivity below that of the uncoated Ti-6Al-4V. While this was not the desired outcome of the analytical approach, it may correlate with real-life conditions where crocodile clips are used.

### 4.2. Numerical Approach

While the analytical calculations used a geometrical model to maintain reasonable mathematical simplicity, the use of numerical methods allowed calculations with a more complex geometry. Since manufacturers anodize pedicle screws with distinct colours to identify their diameter, this means that, experimentally, it would be necessary screws with different diameters to test the effect of increased TiO_2_ layer thickness. By resorting to numerical methods, it was possible to test different thicknesses for the same screw geometry and dimension.

A full-fidelity pedicle screw digital file was used, and the experimental four-point measurement system reported by Fonseca et al. [7] was computationally mimicked. In the numerical analysis, the current and ground terminals had perfect contact with the surface of the screw. While this could result in a lower current density, the use of a constant current terminal guaranteed that the desired current was delivered to the contact terminal. The voltage probes were placed at the middle of the threaded body since this region had a more uniform cross-sectional area. This avoided the slightly conical shape of the screw neck region, which also featured thread initiation.

Similar to the analytical calculations, the model’s conductivity had a small drop. However, it features a substantially lower conductivity, even for the condition where the TiO_2_ layer is absent. This can be attributed to the different geometries of the screws from the current source location down to the section of analysis. The current source was located at the head of the screw, which had a small cross-sectional area. This acts as a “current bottleneck”, resulting in a decrease in the current density at that location, as denoted by Equation (7). This possible effect of morphology correlates with prior observations. While using the same type of monoaxial screw with a crown-type head, Limthongkul et al. [8] applied current with a monopolar probe at the screw-shank connection, while Fonseca et al. [7] used alligator clips on one of the crown’s edges. In the first case, the electrical current crossed the screw through a somewhat uniform cross-section area of its body. In the second case, the initial current path was affected by the crown’s smaller cross-sectional area before reaching the screw’s body, where resistance measurements were performed. This hypothesis may explain why Fonseca et al. [7], when analysing two pedicle screws from the same manufacturer, found a higher conductivity in the one that did not feature a crown-like head as a locking mechanism.

### 4.3. Experimental Approach

Analytical and numerical methods are a good way to control some confounding variables and obtain theoretical results. Nonetheless, experimental analysis allows not only the verification of the theoretical models but also to put into evidence other aspects that may have been left out of consideration.

The pedicle screws used in the experimental analysis were studied “as delivered”, meaning that their properties and, more specifically, the thickness of their oxide layer, were not previously assessed. The pedicle screw colour is an indication of the anodization voltage and the TiO_2_ layer thickness, as this is the result of a light interference effect and may present different hues if an acidic or alkali electrolyte is used [16]. In the case of this study’s specimens, an alkali electrolyte was used, and the theoretical oxide thickness can be extrapolated. However, the lack of information regarding the temperature and anodization duration hinders a more in-depth understanding of the TiO_2_ layer properties, as these parameters can influence the oxide formation rate [43]. 

The middle of the threaded body was chosen for the numerical simulation and experimental measurements. Despite the length and diameter of the screws, the head dimensions are the same for each screw. The screw’s neck presents a transition of the head feature to the threaded body, with a slightly conic shape that reduces rapidly until reaching the threaded body with a more uniform cross-sectional area. By selecting this middle region, the use of a single value for the cross-sectional area was possible.

The experimental results indicate that all analysed screws differ from one another. The notable exceptions are SB—which presents a conductivity identical to DB2 and BG3—as well as DB1 and DB3, which have the same conductivity. This was expected for DB screws because they both have a theoretical anodic layer of 30 nm. However, DB2 differs from the other screws in the group, which denotes the substantial effect that the effective lead distance can imprint on the experimental results. The experimental values seem to indicate that pedicle screws with the same anodization can present different electrical properties. This is most likely attributed to the mechanical wear of the screws due to handling, different positioning of the current alligator clips, and even TiO_2_ layer porosity. However, it is interesting to notice that the numerical model is a digital representation of the DB2 screw, and the section resistances obtained with the numerical and experimental approaches present identical values.

When analysing the effect of the screw group, it becomes evident that each group presents a specific range of conductivities. As observed in the analytical results, thicker TiO_2_ layers present greater resistance, especially in larger-diameter models. This increased resistance, along with the resistance of the Ti-6Al-4V core of the screw, results in a calculated conductivity that clearly decreases with an increase in the theoretical TiO_2_ thickness and, therefore, the anodization voltage. Additionally, the total screw resistance also seems to correlate with TiO_2_ layer thickness. According to Equation (7), the resistance of the screw should increase with its length or with shorter diameters. Screws DG1, DB2, and BG1, have the same length but different diameters. This should have resulted in DG having the smallest resistance because it has the largest diameter, and DB2 has the highest resistance. However, the experimental results indicate the contrary, which correlates with the lower conductivity observed. Wang et al. [40] also demonstrated that hollow screws, which have less conductive material, result in higher resistances than same-diameter solid screws.

### 4.4. Practical Implications

While the results reported in the literature are not entirely conclusive and in agreement between approaches, some relevant aspects were highlighted in this study. For instance, an analytical approach revealed that the TiO_2_ layer presents a substantial radial resistance with an increase in its thickness. This means that even if the pedicle screw core is an ideal conductor and in the vicinity of a nerve root, it will still require higher current levels to elicit a nerve response.

Despite the increased TiO_2_ layer resistance with its thickness, all the approaches presented in this study do not indicate that increased TiO_2_ layer thickness will result in a practical impact on the electrical current stimulation of pedicle screws. In the numerical approach, the terminal voltage reflects the compliance voltage that a constant current source requires to drive the desired 10 mA current through the pedicle screw. This statement is true when having a ground terminal in direct contact with the pedicle screw. If the screw is embedded in bone, a larger resistance will be present between the screw and ground due to the added resistance of biological tissues. According to the estimation of Norton et al. [9], the added resistance of bone, muscle, and fat (but not fluids) can be as high as 21.3 kΩ. This means that the 25.1 Ω screw resistance obtained at a 200 nm layer thickness would only represent 0.1% of the system’s total resistance. For a 10 mA stimulation current, this would require a compliance voltage of 213 V, well within most high-voltage electrical stimulators’ ability. Indeed, currents up to 18 mA may be used without the risk of reaching the maximum 400 V compliance voltage of devices such as the Digitimer DS7A (Digitimer, Welwyn Garden City, UK). This may not even be necessary, as current evidence suggests that stimulation currents below 10 mA have a higher diagnostic odds ratio [5]. The experimental results also demonstrate that the total resistance of the pedicle screws, even those with a larger theoretical TiO_2_ layer thickness, do not have a high enough electrical resistance to compromise the compliance of the electrical stimulator.

All these results and subsequent observations support that the electrical properties of pedicle screws are not significantly affected by the insulating properties of the surrounding TiO_2_ layer. It also highlights that the resistance of the screw is proportionally small in comparison to the resistance of the surrounding tissues and therefore may not be the most preponderant variable during triggered electromyography. Nevertheless, experimental studies are still required to ensure that real-life testing conditions do reflect the same results. 

In the presence of an electrolyte such as bodily fluids, the TiO_2_ layer can create a capacitive effect [44], which results in a high reactance at moderate to high frequencies. The extent of this effect on the ability to conduct electrical stimuli has not been extensively addressed in the scientific literature. However, Donohue et al. [4] has reported alterations in the time-current and frequency spectrum of the stimulus applied to pedicle screws with different titanium alloys. In the present study, only direct current tests were performed, meaning that future studies should also consider the current step response and the effect of alternating current on pedicle screw impedance.

### 4.5. Limitations

While the analytical study provides an order of magnitude of the resistance and conductivity, and how the interaction of the material may influence the overall electrical response, it fails to take into consideration many other effects. For instance, the quantum interaction of the electrons, the semi-conductive nature of TiO_2_ and the presence of dopants, or the precise chemical composition of the TiO_2_ layer are not easily taken into consideration. The surface morphology of the TiO_2_ layer was also not entirely considered, with aspects such as grain size, porosity, and other irregularities not being considered in calculations. Another limitation of the analytical study is that it is based on a simplified model that does not reproduce all the geometrical features of a pedicle screw. 

To improve on this last topic, a numerical analysis was performed. Although an analysis section of 10 mm was selected as an arbitrary length to facilitate calculations, a 9 mm length might have been a better choice since this is a multiple of the thread pitch, thus ensuring that the mass of three full revolutions of the screw helical pattern was represented in the calculation. Nevertheless, the cross-sectional area of the screws was identical throughout the length of the analysis section. This is a feature of a helical pattern, which replicated the same cross-section, albeit with different rotations, along its axis. This was taken into consideration for the calculations. The main limitation of the experimental approach, in addition to the length of the section of analysis and its manual application, relies on the unknown composition of the TiO_2_ layer and its effective thickness. However, without access to specific equipment to perform these analyses, we had to rely on available literature and reasonable approximations.

## 5. Conclusions

Both analytical and numerical approaches have demonstrated that the TiO_2_ layer coating pedicle screws is a source of high electrical resistance. However, due to the apparent parallel arrangement of its resistance with that of the inner Ti-6Al-4V core, the total conductivity is not significantly affected. In fact, the numerical analysis demonstrated that even at a higher layer thickness, the total screw resistance did not increase to values that would compromise the ability of a conventional high-voltage constant current source delivery of a 10 mA electrical stimulus. This seems to indicate that anatomical considerations, such as the transverse electrical resistance of the body, may be a more preponderant aspect to consider. The point of application of the electric current should be chosen with care since the screw design may affect the ability of electrical current to flow from the point of application. 

Nevertheless, the experimental study demonstrated that pedicle screws’ electrical properties differ in function of the TiO_2_ layer thickness, denoting that pedicle screws are not all electrically identical. Considering the controversy regarding the pedicle screw’s electrical characteristics, further studies on the TiO_2_ coating should be pursued. This is particularly important considering the potential capacitive effect that may occur when these screws are implanted in a highly ionic conductive medium. A better understanding of pedicle screw electrical characteristics may allow for the development of new stimulation techniques.

## Figures and Tables

**Table 1:** Physical characteristics of the selected pedicle screws and their division into anodization groups.

		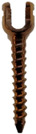	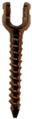	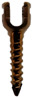	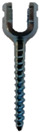	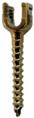	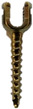	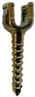	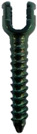
		Group I			Group II	Group III			Group IV
Name		DB1	DB2	DB3	SB	BG1	BG2	BG3	DG
Anodization Voltage (V)		15			35	60			75
Theoretical TiO_2_ thickness (nm)		30			75	120			150
Nominal Diameter (mm)		6.5			4.5	5.5			7.5
Cross-sectional Area (mm^2^)		24.50			11.74	17.54			32.61
Head-to-tip length (mm)		60	55	45	50	55	50	45	55
Thread length (mm)		45	40	30	35	40	35	30	40

**Table 2:** Electrical properties of the pedicle screws obtained through numerical methods.

TiO_2_ Thickness (nm)	Terminal Voltage (mV)	Terminal Resistance (mΩ)	Section Resistance (mΩ)	Calculated Conductivity (S/m)
0	0.03	2.58	0.8405	485,615
5	6.31	630	0.8494	480,537
25	31.4	3140	0.8494	480,509
50	62.8	6280	0.8496	480,429
75	94.2	9420	0.8499	480,254
100	125.6	12,560	0.8501	480,119
125	157.0	15,700	0.8504	479,955
150	188.3	18,830	0.8515	479,374
175	219.7	21,970	0.8522	478,947
200	251.1	25,110	0.8525	478,778

**Table 3:** Electrical properties of the pedicle screws obtained through the experimental protocol.

Group	Screw	Total Resistance (mΩ)	Section Parameters			Screw Results		Group Results	
			Cross-Sectional Area (mm^2^)	Effective Lead Distance (mm)	Resistance (mΩ)	Calculated Conductivity (MS/m)	95% CI	Calculated Conductivity (MS/m)	95% CI (MS/m)
I	DB1	62.6 ± 2.1	24.50	10.19	0.74 ± 0.05	0.564 ± 0.041	[0.564; 0.565]	0.556 ± 0.037 ^2,3,4^	[0.555; 0.557]
	DB2	48.4 ± 4.8		10.84	0.82 ± 0.05	0.540 ± 0.030 *	[0.539; 0.540]		
	DB3	17.4 ± 1.1		10.19	0.74 ± 0.06	0.564 ± 0.041	[0.563; 0.564]		
II	SB	73.4 ± 3.0	11.74	10.04	1.56 ± 0.05	0.549 ± 0.019	[0.548; 0.549]	0.549 ± 0.019 ^1,3,4^	[0.548; 0.550]
III	BG1	239.2 ± 1.9	17.54	9.41	1.04 ± 0.05	0.517 ± 0.026 *	[0.517; 0.518]	0.533 ± 0.026 ^1,2,4^	[0.532; 0.533]
	BG2	119.6 ± 1.9		12.10	1.30 ± 0.00	0.531 ± 0.001 *	[0.530; 0.532]		
	BG3	59.6 ± 2.1		9.22	0.96 ± 0.05	0.549 ± 0.032 *	[0.548; 0.550]		
IV	DG	259.4 ± 3.6	32.61	9.64	0.60 ± 0.00	0.492 ± 0.000	[0.492; 0.493]	0.492 ± 0.000 ^1,2,3^	[0.492; 0.493]

Note: Significant differences between screws in the same group are identified with an asterisk (*). Superscript numbers denote a difference in comparison to the corresponding group number.

## Data Availability

The data generated for this work are available upon reasonable request from the corresponding author.
